# Tackling Critical Catalytic Residues in *Helicobacter pylori* l-Asparaginase

**DOI:** 10.3390/biom5020306

**Published:** 2015-03-27

**Authors:** Maristella Maggi, Laurent R Chiarelli, Giovanna Valentini, Claudia Scotti

**Affiliations:** 1 Unit of Immunology and General Pathology, Department of Molecular Medicine, University of Pavia, Via Ferrata 9, Pavia 27100, Italy; E-Mail: maristella.maggi01@universitadipavia.it; 2 Laboratory of Protein Biochemistry, Department of Biology and Biotechnologies “L. Spallanzani”, University of Pavia, Viale Taramelli 3/B, Pavia 27100, Italy; E-Mail: giovanna.valentini@unipv.it; 3 Laboratory of Molecular Microbiology, Department of Biology and Biotechnologies “L. Spallanzani”, University of Pavia, Via Ferrata 9, Pavia 27100, Italy; E-Mail: laurent.chiarelli@unipv.it

**Keywords:** *Helicobacter pylori*, l-asparaginase, rational engineering, active site mutants, acute lymphoblastic leukemia, catalytic triad residue role

## Abstract

Bacterial asparaginases (amidohydrolases, EC 3.5.1.1) are important enzymes in cancer therapy, especially for Acute Lymphoblastic Leukemia. They are tetrameric enzymes able to catalyze the deamination of l-ASN and, to a variable extent, of l-GLN, on which leukemia cells are dependent for survival. In contrast to other known l-asparaginases, *Helicobacter pylori* CCUG 17874 type II enzyme (HpASNase) is cooperative and has a low affinity towards l-GLN. In this study, some critical amino acids forming the active site of HpASNase (T16, T95 and E289) have been tackled by rational engineering in the attempt to better define their role in catalysis and to achieve a deeper understanding of the peculiar cooperative behavior of this enzyme. Mutations T16E, T95D and T95H led to a complete loss of enzymatic activity. Mutation E289A dramatically reduced the catalytic activity of the enzyme, but increased its thermostability. Interestingly, E289 belongs to a loop that is very variable in l-asparaginases from the structure, sequence and length point of view, and which could be a main determinant of their different catalytic features.

## 1. Introduction

*Helicobacter pylori* l-asparaginase (HpASNase; amidohydrolases, EC 3.5.1.1) is a type II l-asparaginase. The enzyme is capable of carrying out both l-asparagine (l-ASN) and l-glutamine (l-GLN) deamination with a stronger preference for the former. In contrast to other type II l-asparaginases, HpASNase was found to be cooperative and to have a lower affinity *vs.* l-GLN [1,2]. Such characteristics may suggest different interactions with the substrate with regard to other known l-asparaginases.

Bacterial l-asparaginases, which are an important class of chemotherapeutic drugs [3], are tetrameric enzymes consisting of four identical subunits, each typically including nearly 333 amino acids, organized in dimers of intimate dimers [4]. Each subunit has two distinct α/β domains, a larger N-domain and a smaller C-domain, connected by a short loop. The intimate dimers are defined by A/C and B/D monomers contacts [5]. The enzyme has four active sites located at the intimate dimers interface and consisting of two groups of residues belonging to the N- and C-domain, respectively, of the two facing subunits [6,7,8]. The N-domain participates in active site formation with two distinct structural elements, a rigid one and a flexible one (namely, the active site flexible loop). The latter is also involved in regulating binding site accessibility and, after substrate binding, it re-locates on the active site for the correct localization of the catalytic residues [4].

The catalytic mechanism of l-asparaginases depends on two highly conserved catalytic triads consisting of Thr-Tyr-Glu and Thr-Asp-Lys [7,9,10,11,12] represented by T16-Y29-E289 and T95-D96-K168 in *Helicobacter pylori* l-asparaginase [11]. In a first step of the reaction, the activated Thr of the first triad operates a nucleophile attack on the C atom of the substrate amide, resulting in the acyl-intermediate form of the enzyme. The latter is resolved by a water molecule nucleophile attack on the same atom. The Thr of the second triad is very likely responsible of this water molecule activation [7,11]. Within this simple two-steps mechanism the role of each residue in catalysis and substrate binding needs to be investigated further.

Moreover, despite their highly similar structural architecture, type II l-asparaginases from different microbial sources show different kinetics towards the two substrates. Particularly, *Helicobacter pylori* CCUG 17874 l-asparaginase (HpASNase), differently from other type II ASNases, has a quite similar catalytic activity *vs.* l-ASN and l-GLN but an almost 150 times higher affinity for the former substrate [1]. Interestingly, the HpASNase is also the only type II l-asparaginase with a cooperative kinetic *vs.* l-GLN. The explanation of such behavior can rely on structural differences between HpASNase and other type II l-asparaginases, most of all in intersubunits interactions at the intimate dimer interface. Analysis of the HpASNase highly homologous (92% sequence identity) *Helicobacter pylori* J99 l-asparaginase (HpA) structure highlights some specific peculiarities, mostly in the flexible loop (residues 19–46) and in the 286–297 loop, which may explain the HpASNase peculiar catalytic properties [11].

In this study, some critical amino acids forming the active site of HpASNase (T16, T95 and E289) have been tackled by rational engineering in the attempt to better define their role in catalysis and to achieve a deeper understanding of the peculiar cooperative behavior of this enzyme. Particularly, we focused on residues belonging to the active site that are highly conserved in l-asparaginases (T16 and T95) and/or laying on structural elements that mostly differ from other l-asparaginases. In fact, residue E289 belongs to the 286–297 loop that in HpA is closer to the active site than in other type II l-asparaginases. Because of its position, E289 is involved in substrate binding and intersubunits interactions and therefore it can play a unique role in HpASNase catalytic properties.

## 2. Results

In our work we aimed at understanding the peculiar kinetic behavior of HpASNase by a deep analysis of its active site. In order to do this, we subjected the HpASNase gene to site direct mutagenesis to obtain T16E, T95D and E289A replacements. The replaced amino acids are part of the active site and, interestingly, E289, a subunits interface residue, belongs to a loop that differs for its spatial localization from other bacterial l-asparaginases. Sequencing of the mutated insert revealed the presence of the desired replacement in all the cases except for a random replacement at position n.283 (C > G) resulting, instead of the planned T95D, in the amino acid replacement T95H. Beyond T95D, also the serendipitous T95H mutant was characterized. In fact, mutation T95H may strongly perturb the enzyme active center both directly and at long range, altering enzyme catalysis and/or substrates binding. Moreover, the T95H mutant structural model shows a delocalization of K168, a residue of the first catalytic triad (See Discussion Section).

### 2.1. Steady-State Kinetics of H. Pylori l-asparaginase Variants

**Figure 1:**
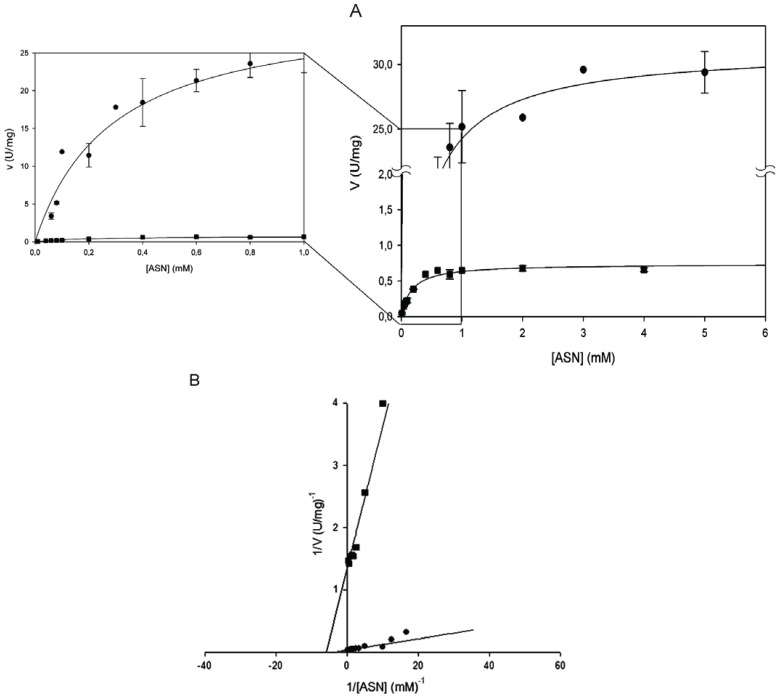
Steady-state kinetics of HpASNase E289A mutant as a function of l-asparagine (l-ASN) compared to wild type enzyme. Activity dependence on substrate concentrations was measured at 37 °C in a reaction mixture containing 50 mM 4-(2-Hydroxyethyl)piperazine-1-ethanesulfonic acid (HEPES) buffer pH 7.5, 1 mM α-ketoglutarate (α-KG), 0.24 mM NADH, 20 U l-glutamate dehydrogenase and 0 to 10 mM l-ASN, in a final volume of 0.5 mL. Panel A represents data as Michaelis-Menten plot, with the off-set panel showing a detail of the plot corresponding to low l-ASN concentrations (0–1 mM). Panel B represents data as Linweaver-Burke plot. Wild type ●, E289A mutant ■. Data are represented as averages (*n* ≥ 3) with error bars (SE).

Kinetic parameters were determined by Lineweaver-Burk plot and found to be identical by the non- linear regression fit of the Michaelis-Menten plot (Table 1). All the amino acid replacements resulted in a massive or complete catalytic activity impairment. Mutants T16E, T95D and T95H showed to be completely devoid of activity towards both l-ASN and l-GLN. Their quaternary structure folding was verified by analytic gel-filtration and the profiles were not significantly different from wild type [13,14]. The mutant enzyme E289A was, instead, catalytically active, though only toward l-ASN and with a 40-fold reduced k_cat_ (0.44 s^−1^ *vs.* 19.26 s^−1^ of the wild type; Figure 1B and Table 1).The kinetic behavior resembles the hyperbolic one of the wild type enzyme (n_H_ = 1) and the K_m_ was almost unchanged (0.17 mM *vs.* 0.29 of wild type; Figure 1B and Table 1).

**Table 1:** Apparent kinetic constants of *Helicobacter pylori* CCUG 17874 type II enzyme (HpASNase) wild type and mutant forms.

Enzyme	l-Asparagine				l-Glutamine			
	k_cat_ (s^−1^)	S_0.5_ (mM)	k_cat_/S_0.5_ (s^−1^ mM^−1^)	n_H_	k_cat_ (s^−1^)	S_0.5_ (mM)	k_cat_/S_0.5_ (s^−1^ mM^−1^)	n_H_
wild type	19.26 ± 0.56	0.29 ± 0.03	66.40	1.0 ± 0.06	22.10 ± 1.39	46.4 ± 4.02	0.47 ± 0.10	2.0 ± 0.10
T16E	ND	ND	ND	ND	ND	ND	ND	ND
T95D	ND	ND	ND	ND	ND	ND	ND	ND
T95H	ND	ND	ND	ND	ND	ND	ND	ND
E289A	0.44 ± 0.01	0.17 ± 0.02	2.60	1.0 ± 0.05	ND	ND	ND	ND

Results are means (±SE) of 3 determinations from at least 3 protein preparations. ND: None Detected.

### 2.2. Thermostability of H. pylori l-asparaginase Variants

**Figure 2:**
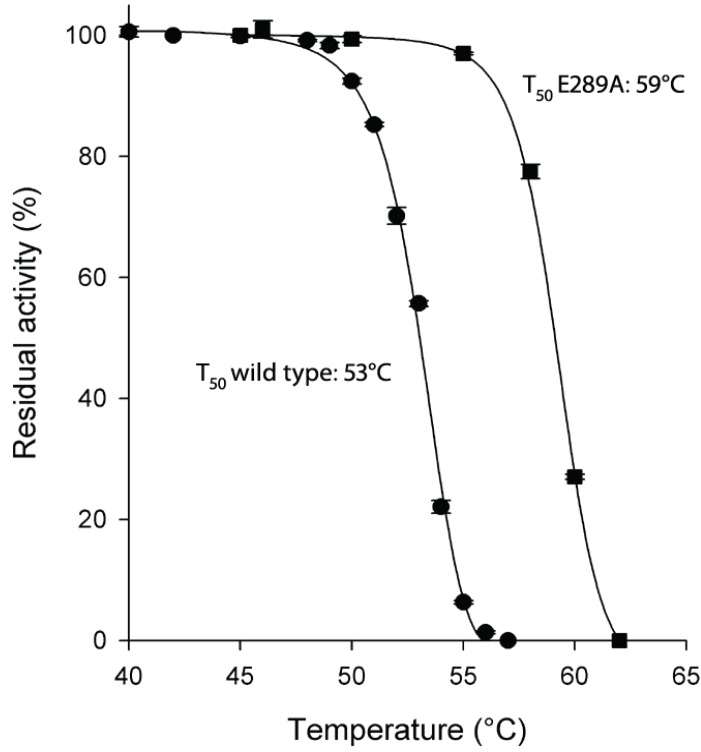
Thermostability of HpASNase E289A mutant compared to wild type enzyme. Plot of the residual activities at 10 min heat-treatment *vs.* temperatures. Incubation buffer was 20 mM Na-phosphate pH 7.5. After 10 min incubation at a given temperature, the enzyme sample was chilled and the residual activity measured. T_50_ is the incubation temperature at which the enzyme loses 50% of its activity in 10 min. Wild type ●, E289A mutant ■. Data are represented as averages (*n* ≥ 2) with error bars (SE).

As for protein stability, the E289A mutant enzyme resulted to be very thermostable with a T_50_ value 6 °C higher than the wild type enzyme (59 °C *vs.* 53 °C of wild type; Figure 2).

## 3. Discussion

*Helicobacter pylori* CCUG 17874 wild type recombinant enzyme has been previously characterized from the biochemical point of view [1,2] and presents kinetic and protein stability features quite different from other reported bacterial l-asparaginases. The enzyme has a hyperbolic response to l-ASN as other type II l-asparaginases [9], but, as opposed to other type II l-asparaginases, HpASNase exhibits a sigmoidal response to l-GLN. Moreover, presently, HpASNase is the only l-asparaginase with comparable turn over numbers for both substrates, but quite different apparent affinity for them [3]. In fact, the wild type enzyme has an affinity nearly 150 times higher for l-ASN than for l-GLN. Considering the peculiar characteristics of HpASNase, we studied the role of some of the active site forming residues using rational engineering. In HpASNase, the catalytic triads are constituted by residues T16-Y29-E289 and T95-D96-K168. Given the essential role of the threonines in l-asparaginases catalysis, we replaced T16 with a glutamate and T95 with an aspartate. The serendipitous mutation T95H was also characterized, for its potential to disrupt the three-dimensional configuration of the active-site.

**Figure 3:**
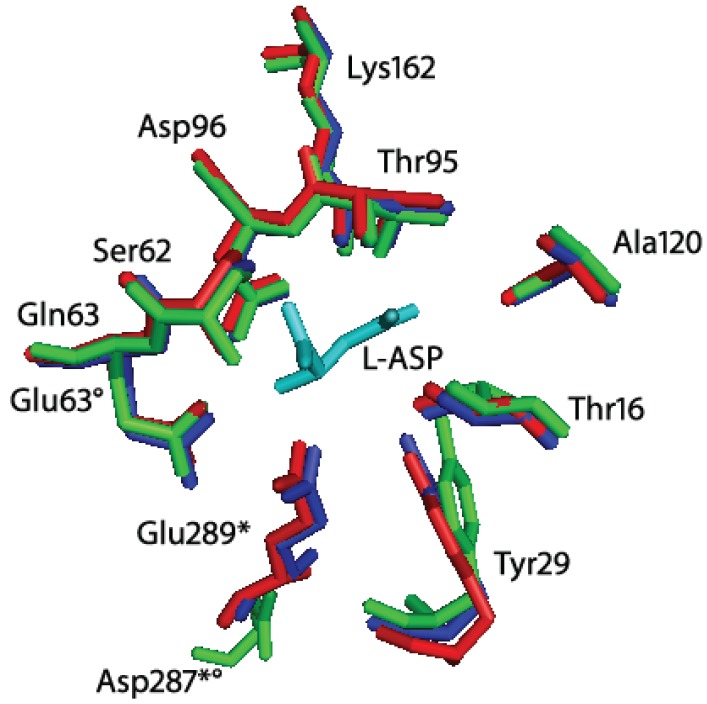
Superimposition of *Helicobacter pylori* J99 (HpA, PDB ID: 2WLT, red), *Escherichia coli* (EcA, PDB ID: 3ECA, blue) and *Erwinia chrysanthemi* (EwA, PDB ID: 1JSR, green) l-asparaginases active site residues. In cyan: l-Asp (reaction product). The residues are labeled according to the *H. pylori* enzyme residues number. * indicates residues of the second subunit of the intimate dimer; indicates residues that are not conserved in *Erwinia chrysanthemi* l-asparaginase. Besides the presence of a Glu instead of a Gln in position 63, the main structural difference between HpA and EwA is the localization of D287 in EwA with respect to the corresponding E289 in HpA. The remaining active site residues are quite conserved in all the three enzymes.

From a structural point of view, the comparison of the highly homologue HpA (*Helicobacter pylori* J99 type II l-asparaginase, PDB ID: 2WLT, [11]; identity to HpASNase: 92%) with other bacterial l-asparaginases underlined a different spatial disposition of residues 210–218 and of the 286–297 loop (Figure 3). The latter comprises the triad residue E289 that in HpASNase results to be quite close to the active site [11]. We reasoned that the different E289 localization could be an explanation of HpASNase cooperativity toward l-GLN, being the residue at the intimate dimer interface and acquiring interactions different from the same residue in other type II l-asparaginases.

**Figure 4:**
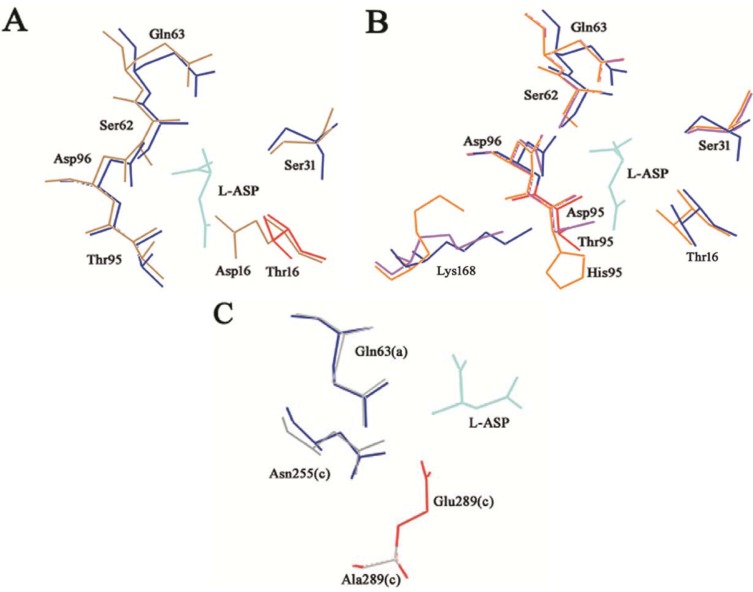
T16E, T95D, T95H and E289A HpASNase mutants’ structural models. The mutants models are superposed onto the HpA wild type crystal structure (blue lines, PDB ID: 2WLT [11]) and the native (replaced) residues are shown in red. Lower case letters indicate the subunit. The bonded product l-ASP is reported in all the models and represented as cyan lines. (**A**) T16E mutant model represented as sand lines. Residues S31, Q63, S62, D96 and T95 are as well reported being involved in structural interactions with the T16 residue. (**B**) T95D (pink lines) and T95H (orange lines) structural models. Residues T16, S31, Q63, S62, D96 and K168 are as well reported being involved in structural interactions with the T95 residue. (**C**) E289A (grey lines) structural model. Residues Q63 and N255 are as well reported being involved in structural interactions with the E289 residue.

T16 β-OH is the nucleophile group responsible for the first attack to the C atom of the substrate amide that results in the release of ammonia and in the formation of the enzyme acyl-intermediate. As expected, its replacement with an Asp, a large and charged residue, resulted in a complete loss of activity. Very likely, the Asp δ COO^−^ group, although in principle may act as a nucleophile group to trigger the enzyme acyl-intermediate formation, may be too far from the substrate and unable to participate in the nucleophile attack needed for the first step of the catalysis (Figure 4A).

T95 does not directly participate in catalysis, but it is involved in the process of activation of the water molecule responsible for the second nucleophile attack during the deacylation reaction. Moreover, T95 participates in substrate binding. Replacements of T95 with a histidine or an aspartate completely impaired enzyme catalysis. The Asp strongly negative charge in position 95 can interfere with the proper localization of the nucleophile water molecule. Above all, residue 95 participates in the hydrogen-bonding network along with the other two triad residues (D96 and K168). In the case of T95H and T95D mutants, the presence of a larger or a charged residue, respectively, may compromise the proper catalytic triad organization, affecting mostly the K168 side chain localization (Figure 4B).

Residue E289 belongs to the 286–297 loop that is very variable in position in l-asparaginases. Crystallographic evidences showed that in HpA the loop is closer to the active site, especially with respect to the EwA (*Erwinia chrysanthemi* type II l-asparaginase) enzyme [11,15]. In the HpASNase model, E289 locates around the active site, and in proximity of Q63 from the nearby monomer and N255 from the same monomer. It has been suggested the involvement of all the three residues in regulating the kinetic properties of HpASNase enzyme, in substrate binding and inter-subunits interactions. In order to better understand the actual role of E289 in HpASNase kinetic properties, we replaced it with an alanine causing the loss of interactions in the intersubunit space and with the substrate. Moreover, the alanine in position 289 should lose all the interactions with N255 and Q63, possibly resulting in a loop re-localization in a conformation more similar to the EwA enzyme. Actually, in the EwA structure the residue corresponding to N255 is a Ser, which shows to be unable to interact with the triad Glu and the near monomer Asp (Figure 4C) [11]. The HpASNase E289A recombinant enzyme resulted to have very low activity toward l-ASN (40-fold reduced k_cat_) and to be practically devoid of activity toward l-GLN. Interestingly, the K_m_ value *vs.* l-ASN was almost unchanged, suggesting that the E289 residue has a marginal role in substrate binding, whereas its catalytic role and its intersubunit interactions are needed for proper catalysis in HpASNase, as hypothesized on the basis of structural data. Moreover, the dramatic loss of activities due to the E289 removal suggests that interactions between the 286–297 loop and the active site of HpASNase are essential in order to properly coordinate both substrate and catalytic residues, a feature not shared with other type II l-asparaginases (like EwA), where such interactions are absent [11].

Although the E289A mutant presented a dramatically reduced catalytic activity, the enzyme resulted to be very thermostable. The naturally occurring E289 residue is solvent accessible and locates at the intimate dimer interface. Replacement of E289 with an alanine removes the canonical intersubunit ionic interactions and most likely gives rise to a new set of interactions leading to a more rigid and stable enzyme conformation. Moreover, this new conformation could be the reason of the inability of the mutant to perform the correct catalysis.

## 4. Experimental Section

### 4.1. Generation, Expression and Purification of HpASNase Mutants

To obtain enzymatic variants of *H. pylori* CCUG 17874 l-asparaginase, the pDC1 plasmid derived from pET101 D-TOPO plasmid (Invitrogen, Waltham, MA, USA) and containing the *ansB* wild type gene [1] was subjected to site direct mutagenesis. Briefly, reactions were carried out in 1X *PfuUltra* buffer, 0.1 mM dNTPs, 0.5 µM each sense and anti-sense primers, 2.5 ng template dsDNA and 2.5 U *PfxUltra* (Invitrogen) in a final volume of 50 µL. PCR products were checked on a 1% agarose gel and digested with *Dpn* I restriction enzyme (10 U, New England BioLabs, Ipswich, MA, USA) at 37 °C for 1 h in order to remove all the native DNA before transformation. The oligonucleotides used as mutagenesis primers are reported in Table 2. All the mutants were verified by insert sequencing.

*E. coli* BL21(DE3) p*LysS* cells transformed with plasmid pDC1 mutant derivatives were grown in a ZYP-5052 medium (10 g/L Tryptone, 5 g/L Yeast Extract, 50 mM Na_2_HPO_4_, 50 mM KH_2_PO_4_, 25 mM (NH_4_)_2_SO_4_, 1 mM MgSO_4_, 0.5% w/v glycerol, 0.05 w/v glucose, 0.2% w/v α-lactose) containing 100 μg/mL ampicillin and protein expression was performed through auto-induction according to Studier [16].

Cells harvested from 1 L culture broth were suspended in 50 mM Na-phosphate pH 8.0, 300 mM NaCl, 10 mM imidazole (ImOH) added with 1 mM PMSF, sonicated and centrifuged. HpASNase recombinant variants were purified following the procedure used for the wild type enzyme [1]. The fractions obtained were assayed for asparaginase activity and checked for homogeneity by SDS-PAGE on a 12% gel. The purified fractions positive for HpASNase and pure were pooled together. Protein concentration was determined according to Lowry *et al.* [17].

**Table 2:** Sense and anti-sense oligonucleotides used for mutagenesis experiments on the *ansB* wild type gene. Bold underlined letters represent replaced nucleotides.

Mutant	Oligonucleotides	Gene Replacement
T16E	Forward 5'-GACAGGGGGGGAGATTGCAGGGAGTG-3';	c.46(5'-ACG-3' > 5'-GAG-3')
	Reverse 5'-CTGTCCCCCCCTCTAACGTCCCTCAC-3'	
T95D	Forward 5'-GTCATCACGCATGGCGACGACACTTTAGAAGAG-3';	c.283(5'-ACG-3' > 5'-GAC-3')
	Reverse 5'-CAGTAGTGCGTACCGGTCCTGTGAAATCTTCTC-3'	
E289A	Forward 5'-GAGATTACTTCAGGCGCGATTGATGACAAGACC-3';	c.865(5'-GAG-3' > 5'-GCG-3')
	Reverse 5'-CTCTAATGAAGTCCGCGCTAACTACTGTTCTGG-3'	

### 4.2. l-asparaginase and l-Glutaminase Activity Assay and Kinetic Analysis

Both l-asparaginase and l-glutaminase activities were determined at 37 °C with l-ASN or l-GLN, respectively, as a substrate, by l-glutamate dehydrogenase coupled spectrophotometric assay according to Balcão [18] and Derst [19] and in the same conditions of the wild type enzyme [1]. Briefly, the standard reaction mixture contained 50 mM 4-(2-Hydroxyethyl)piperazine-1-ethanesulfonic acid (HEPES) buffer pH 7.5, 1 mM α-ketoglutarate (α-KG), 0.24 mM NADH, 20 U l-glutamate dehydrogenase (in large excess, in order to avoid a rate limitation effect) and 5 mM l-ASN or 150 mM l-GLN, in a final volume of 0.5 mL. The reaction was started by adding enzyme solution (5–50 μg) and NADH consumption was monitored at 340 nm with a Jasco V-550 UV/VIS spectrophotometer (Jasco-Europe, Cremella, Italy).

For steady-state kinetic studies, enzyme activity was assayed in triplicate under the above conditions using at least 10 different l-ASN or l-GLN concentrations (0–10 mM l-ASN or 0–400 mM l-GLN). The reaction was started by adding the substrate to the reaction mixture containing 5–50 μg enzyme solution. The kinetic parameters V_max_ and K_m_ were determined both by Lineweaver-Burk and by Michaelis-Menten plots using the Enzyme Kinetic Module 1.1 for Sigma Plot (IBM SPSS Inc., Chicago, IL, USA).

k_cat_ or turn-over number, is the number of catalytic events per second. K_m_ is the substrate concentration at which the reaction speed is half maximal. k_cat_/K_m_ is an index of catalytic efficiency at sub-saturating substrate concentration.

One Unit is defined as the amount of enzyme needed to convert 1 µmole of substrate in product in the above described conditions.

### 4.3. Thermal Stability Assay

A parameter useful to determine protein stability is T_50_. To evaluate the stability of HpASNase variants by T_50_, enzyme solutions (0.5–1.0 mg/mL) were incubated at different temperatures in 20 mM Na-phosphate pH 7.5 for 10 min. After incubation, the enzyme solution was chilled on ice and the activity was spectrophotometrically assayed. The residual activity was expressed as the percentage of activity of heat-treated enzyme with respect to the untreated one.

### 4.4. Structural Modeling

For structural modeling of T16E, T95D, T95H and E289A HpASNase variants the released *Helicobacter pylori* J99 structure (PDB ID: 2WLT) was used as a template. Modeling was performed using Modeler 9.13 (San Francisco, CA, USA) [20].

## 5. Conclusions

In this work, we have investigated the role of three catalytic residues in HpASNase enzymatic activity. Our data demonstrate once more an essential role for both triad threonines T16 and T95 in asparaginase and glutaminase activity, a feature shared with all other members of the same class. The present data, in fact, resemble the characteristics of other two HpASNase mutants with amino acid replacement on the same residues (*i.e.*, T16D and T95E [21]), and those of a previously reported *E. coli* ASNase T89V mutant [22]. Because of its localization in the 286–297 loop, we designed the E289A mutation in order to analyze its influence on the peculiar cooperative behavior of the enzyme *vs.* l-GLN. No information could be derived in this respect, as this residue was found to be strictly needed for catalytic activity *vs.* l-GLN. However, the loss of glutaminase activity together with the dramatic reduction in asparaginase activity, strongly suggest that the localization of the 286–297 loop in HpASNase near the active site allows for interactions which are required for the enzyme proper catalysis. This is the first time that kinetic data confirm the catalytic role of this structural element that differs among asparaginases. Further dissection of this feature could help in better understanding their different kinetic properties. A totally unexpected result lies in the increased thermal stability of the E289A mutant, which suggests a stabilizing role for this mutation and, in reverse, a potential role for destabilization in catalysis, a feature which would also deserve further investigation.
